# Association of Retinal Nerve Fiber Layer Thickness, an Index of Neurodegeneration, With Depressive Symptoms Over Time

**DOI:** 10.1001/jamanetworkopen.2021.34753

**Published:** 2021-11-16

**Authors:** Frank C. T. van der Heide, Indra L. M. Steens, Anouk F. J. Geraets, Yuri D. Foreman, Ronald M. A. Henry, Abraham A. Kroon, Carla J. H. van der Kallen, Thomas T. van Sloten, Pieter C. Dagnelie, Martien C. J. M. van Dongen, Simone J. P. M. Eussen, Tos T. J. M. Berendschot, Jan S. A. G. Schouten, Carroll A. B. Webers, Marleen M. J. van Greevenbroek, Anke Wesselius, Annemarie Koster, Nicolaas C. Schaper, Miranda T. Schram, Seb Köhler, Coen D. A. Stehouwer

**Affiliations:** 1 Cardiovascular Research Institute Maastricht School for Cardiovascular Diseases, Maastricht University, the Netherlands; 2 Department of Internal Medicine, Maastricht University Medical Center+, the Netherlands; 3 Department of Psychiatry and Neuropsychology, Maastricht University Medical Center+, Maastricht, the Netherlands; 4 School of Mental Health and Neuroscience, Maastricht University Medical Center+, Maastricht, the Netherlands; 5 Heart and Vascular Center, Maastricht University Medical Center+, Maastricht, the Netherlands; 6 Care and Public Health Research Institute, Maastricht University, the Netherlands; 7 Department of Epidemiology, Maastricht University, the Netherlands; 8 University Eye Clinic Maastricht, Maastricht University Medical Center+, Maastricht, the Netherlands; 9 Department of Ophthalmology, Canisius-Wilhelmina Ziekenhuis Nijmegen, the Netherlands; 10 Department of Complex Genetics and Epidemiology, School for Nutrition and Translational Research in Metabolism, Maastricht University, Maastricht, the Netherlands

## Abstract

**Importance:**

Whether neurodegeneration contributes to the early pathobiology of late-life depression remains incompletely understood.

**Objective:**

To investigate whether lower retinal nerve fiber layer (RNFL) thickness, a marker of neurodegeneration, is associated with the incidence of clinically relevant depressive symptoms and depressive symptoms over time.

**Design, Setting, and Participants:**

This is a population-based cohort study from the Netherlands (The Maastricht Study) with baseline examination between 2010 and 2020 and median (IQR) follow-up of 5.0 (3.0-6.0) years. Participants were recruited from the general population. Individuals with type 2 diabetes were oversampled by design. Data analysis was performed from September 2020 to January 2021.

**Exposures:**

RNFL, an index of neurodegeneration, assessed with optical coherence tomography.

**Main Outcomes and Measures:**

Depressive symptoms were assessed with the Patient Health Questionnaire (PHQ)–9 (continuous score, 0-27) at baseline and over time via annual assessments. The presence of clinically relevant depressive symptoms was defined as a PHQ-9 score of 10 or higher.

**Results:**

We used data from 4934 participants with depressive symptoms over time (mean [SD] age, 59.7 [8.4] years; 2159 women [50.8%]; 870 had type 2 diabetes [20.5%]). Lower RNFL thickness was associated with higher incidence of clinically relevant depressive symptoms (per 1 SD, hazard ratio 1.11; 95% CI, 1.01-1.23) and more depressive symptoms over time (per 1 SD, rate ratio, 1.04; 95% CI, 1.01-1.06), after adjustment for demographic, cardiovascular, and lifestyle factors.

**Conclusions and Relevance:**

The findings of this study suggest that lower RNFL thickness is associated with higher incidence of clinically relevant depressive symptoms and more depressive symptoms over time. Hence, neurodegeneration may be associated with the early pathobiology of late-life depression.

## Key Points

**Question:**

Is neurodegeneration associated with the early pathobiology of late-life depression?

**Findings:**

This population-based cohort study of 4934 participants found that lower retinal nerve fiber layer thickness, an index of neurodegeneration, was significantly associated with higher incidence of clinically relevant depressive symptoms and more depressive symptoms over time, after adjustment for demographic, lifestyle, and cardiovascular risk factors.

**Meaning:**

These findings suggest that neurodegeneration may be associated with the early pathobiology of late-life depression.

## Teaser

This cohort study investigates whether lower retinal nerve fiber layer thickness, a marker of neurodegeneration, is associated with the incidence of clinically relevant depressive symptoms and depressive symptoms over time.

## Introduction

Late-life depression is a debilitating disease that may be caused by neurodegeneration.^1,2^ Clinically, late-life depression is associated with a high comorbidity of psychiatric and physical diseases including neurodegenerative diseases such as Alzheimer disease.^3^ Mechanistically, neurodegeneration in brain regions involved in reward, salience, and cognitive function is thought to lead to disturbances in mood, cognition, and motoric behavior, all of which are clinical symptoms of late-life depression.^1^

Whether neurodegeneration contributes to the early pathobiology of late-life depression remains incompletely understood.^1^ Cerebral neurodegeneration may be a gradual process that starts long before symptoms of late-life depression are detectable.^4^ Biologically, long-term exposure to stress, microvascular dysfunction, amyloid accumulation, and exposure to adverse risk factors such as hyperglycemia are all thought to contribute to deterioration of cerebral neurodegeneration.^1,4^

The retina may provide an opportunity to study the early pathobiology of late-life depression.^5^ In the retina, early neurodegeneration can be assessed as subtle neurodegenerative changes such as retinal nerve fiber layer (RNFL) thinning.^5^ RNFL thinning is thought to reflect loss of retinal ganglion cell axons. Retinal neurodegeneration is a biologically plausible measure of cerebral neurodegeneration because the retina and brain have similar anatomical features and physiological properties.^5^ Indeed, lower RNFL thickness has been associated with magnetic resonance imaging–assessed markers of cerebral neurodegeneration (ie, lower gray and white matter volume),^6^ cognitive decline,^7^ and dementia.^8^

There are few data on the association of RNFL thickness with late-life depression. Some small studies^9,10,11,12^ have found an association between retinal neurodegeneration and depression. However, these studies have important limitations (ie, they used cross-sectional data), they used small clinical study populations, and they did not adjust for potential confounders.^9,10,11,12^

In view of these limitations, the objective of the present study was to investigate whether lower RNFL thickness was associated with incidence of clinically relevant depressive symptoms and depressive symptoms over time by use of data from a large, well-characterized, population-based cohort study. We hypothesized that lower RNFL thickness was associated with greater incidence of clinically relevant depressive symptoms and more depressive symptoms over time.

## Methods

### Study Population and Design

We used data from The Maastricht Study, an observational, prospective population-based cohort study. The rationale and methods have been described previously.^13^ In brief, the study focuses on the causes, pathophysiological processes, complications, and comorbidities of type 2 diabetes and is characterized by an extensive phenotyping approach. All individuals aged 40 to 75 years living in the southern part of the Netherlands were eligible for participation. Participants were recruited through mass media campaigns and from the municipal registries. In addition, to oversample the number of participants with type 2 diabetes for reasons of efficiency, individuals with known type 2 diabetes were recruited via the regional Diabetes Patient Registry via mailings. The present report includes prospective data of 7689 individuals collected between November 2010 and December 2017. Prospective data were available for 6995 participants at 1 year, 6153 participants at 2 years, 5690 participants at 3 years, 5029 participants at 4 years, 4451 participants at 5 years, 2752 participants at 6 years, and 1469 participants at 7 years of follow-up. The baseline examinations of each participant were performed within 3 months.

The study has been approved by the institutional medical ethical committee and the Minister of Health, Welfare and Sports of the Netherlands. All participants gave written informed consent. This study was written in accordance with the Strengthening the Reporting of Observational Studies in Epidemiology (STROBE) reporting guidelines.^14^

### Assessment of Depressive Symptoms and Major Depressive Disorder

Depressive symptoms were assessed with a validated Dutch version of the Patient Health Questionnaire (PHQ)–9 (score range, 0-27).^15^ PHQ-9 data were collected both at baseline and during the 7 years of annual follow-up. The presence of clinically relevant depressive symptoms was defined as a PHQ-9 score of 10 or higher.^16^ Incident clinically relevant depressive symptoms was defined as the absence of clinically relevant depressive symptoms at baseline (PHQ-9 score <10) and the presence of clinically relevant depressive symptoms (PHQ-9 score ≥10) on at least 1 follow-up assessment. More details on the assessment of depressive symptoms are provided in eAppendix 1 in the Supplement.

### Assessment of the RNFL Thickness

We assessed RNFL thickness with optical coherence tomography (Spectralis; Heidelberg Engineering).^17^ The RNFL thickness (micrometers) of both eyes was measured within a 3.45 mm diameter circular scan (12°, 768 voxels, 100 automatic real-time tracking) centered on the optic nerve head. All optical coherence tomography scans were reviewed and their quality was scored. Intra- and interindividual reliability, expressed as intraclass correlation coefficients, were 0.97 and 0.96, respectively.^18^ More details on the assessment of RNFL thickness are provided in eAppendix 1 in the Supplement.

### Assessment of Covariates

As previously described,^13^ we used fasting plasma glucose and 2-hour postload glucose to assess glucose metabolism status according to the World Health Organization 2006 criteria as normal glucose metabolism, prediabetes, type 2 diabetes, type 1 diabetes, or other types of diabetes.^13^ We assessed educational status (low, middle, or high), smoking status (never, current, or former), alcohol consumption (none, low, or high), and partner status (partner or no partner) with questionnaires^13^; assessed medication use via a medication interview; assessed waist circumference (centimeters) and office blood pressure (millimeters of mercury) as part of a physical examination; and determined total cholesterol to high-density lipoprotein ratio in fasting blood samples.^13,19^ More details on the assessment of educational status and the assessment of covariates that were only used in additional analyses are described in eAppendix 1 in the Supplement.

### Statistical Analysis

We inversed (ie, multiplied by −1) RNFL thickness, so that higher values indicate greater neurodegeneration (ie, thinner RNFL). Next, if (for logistical reasons) the RNFL thickness was assessed at a later moment in time than at baseline (190 participants), we used the PHQ-9 score from the annual questionnaire closest in time to the assessment of the RNFL thickness as the baseline PHQ-9 score (so that the time lag between measurements was minimized). Participants were eligible for inclusion in analyses if they did not have clinically relevant depressive symptoms at baseline (ie, PHQ-9 score <10) and if at least 1 PHQ-9 score assessment was available during annual follow-up.

We used Cox proportional hazard regression analyses to study associations of standardized RNFL thickness with the incidence of clinically relevant depressive symptoms (ie, present vs absent) and we expressed results as hazard ratios with corresponding 95% CIs. The date of censoring was defined as the first date at which the PHQ-9 score was 10 or higher (event) or, otherwise, as the last date at which a PHQ-9 assessment was available (if no event had previously occurred). If any annual assessment of PHQ-9 score was missing before the date of censoring, the missing PHQ-9 scores were assumed to be less than 10 (ie, free of clinically relevant depressive symptoms) to maximize the number of participants whose data could be used in the main analyses. In addition, we made a Kaplan-Meier curve to visualize the association between RNFL thickness (entered as tertiles) and incidence of clinically relevant depressive symptoms. Tertiles of RNFL thickness were calculated according to the ordered distribution of RNFL thickness in the study population of the present study.

We used generalized estimating equations analyses (exchangeable correlation structure; negative binomial mode) for analyses of standardized RNFL thickness with depressive symptoms over time (PHQ-9 score 0-27). We used negative binomial regression because the PHQ-9 data are right-skewed (ie, contain many null values). Results were expressed as rate ratios with corresponding 95% CIs.

We checked whether we could assume that results were constant over time (ie, proportional hazard assumption) by testing for interaction with time (ie, entering an interaction term between RNFL thickness and time). If there was no interaction with time, we considered results to be valid over time.

In model 1, we adjusted for age,^20,21^ sex,^21,22^ glucose metabolism status^23,24^ (entered as dummy variables for prediabetes, type 2 diabetes, or other type of diabetes, with normal glucose metabolism status as the reference), and educational status (low [reference], middle, or high).^25,26^ We chose these variables because they are key potential confounders (all) or were oversampled by design (type 2 diabetes). In model 2, we also adjusted for variables of which their status as potential confounder has been less firmly established: waist circumference,^21,27^ total cholesterol to high-density lipoprotein cholesterol ratio,^28,29^ use of lipid-modifying medication (yes vs no),^28,29^ office systolic blood pressure,^28,29^ use of antihypertensive medication (yes vs no),^28,30^ smoking (current, ever, or never [reference]),^21,31^ alcohol consumption (none [reference], low, or high),^29,32^ and partner status (partner vs no partner).^31^

We used interaction analyses to test whether associations differed by sex (ie, between men and women) or glucose metabolism status (ie, between individuals with prediabetes, with type 2 diabetes, or with normal glucose metabolism). Because the number of participants with diabetes types other than type 2 was small, we excluded participants with other types of diabetes from tests of interaction with glucose metabolism status.

To assess the robustness of our findings, we performed several additional analyses. First, we investigated the cross-sectional association between RNFL thickness and the presence of a major depressive disorder (assessed with the Mini-International Neuropsychiatric Interview, the reference standard for the assessment of a major depressive disorder).^33^ The presence of a major depressive disorder was assessed at baseline only. Second, we studied the cross-sectional associations of RNFL thickness with depressive symptoms (continuous) and with the presence of clinically relevant depressive symptoms (present vs absent). Third, we repeated prospective analyses after exclusion of individuals with use of antidepressive medication at baseline, those with a major depressive disorder at baseline, those with an age of onset of major depressive disorder 40 years or younger, and those with more than 2 missing assessments of PHQ-9 data during follow-up.^34^ Last, we performed additional analyses in which we also adjusted for a range of covariates that may be potential confounders (eg, diet and physical activity); we also performed additional analyses in which we excluded individuals with certain retinal diseases (eg, glaucoma) or performed additional analyses in which we replaced certain covariates with other covariates that reflect a similar underling construct (eg, we replaced glucose metabolism status with fasting plasma glucose). More details on additional analyses are presented in eAppendix 1 in the Supplement.

All analyses were performed with SPSS statistical software version 23.0 (IBM SPSS, IBM Corp). For all analyses (including interaction analyses), *P* < .05 was considered statistically significant in 2-sided tests. Data analysis was performed from September 2020 to January 2021.

## Results

### Characteristics of the Study Population

**Figure 1.**
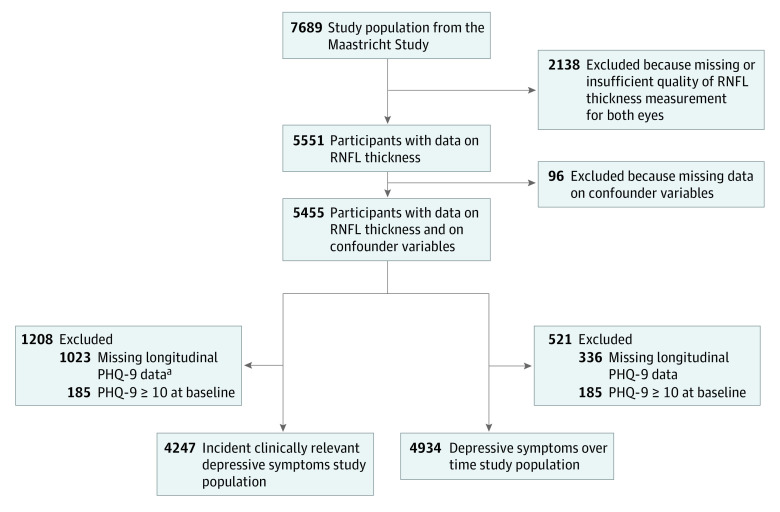
Flowchart for Selection of Participants for Inclusion PHQ-9 indicates Patient Health Questionnaire–9; RNFL, retinal nerve fiber layer. ^a^A total of 1023 participants were excluded because the assessment of clinically relevant depressive symptoms was missing for them at all annual follow-up moments. Of the 4432 participants with data on incidence of clinically relevant depressive symptoms, 3910 participants had complete data at all annual assessments, and 444, 66, and 12 participants had missing data at 1, 3, or 3 moments of annual follow-up, respectively. Of the 4934 participants with prospective data on depressive symptoms, 4822, 4280, 3911, 3467, 2854, 1636, and 710 participants had data on depressive symptoms after 1, 2, 3, 4, 5, 6, or 7 years, respectively, after baseline.

Figure 1 shows a flowchart of the selection of participants for inclusion in analyses. The study populations consisted of 4247 participants for analyses with incident depressive symptoms and 4934 participants for analyses with depressive symptoms over time (mean [SD] age, 59.7 [8.4] years; 2159 women [50.8%]; 870 had type 2 diabetes [ 20.5%]).

Table 1 and eTable 1 in the Supplement show general characteristics of the participants. Participants with a thinner RNFL at baseline were more often men (lowest vs highest tertile of thickness, 799 men [56.5%] vs 625 men [44.1%]) and had a more adverse cardiovascular risk profile; individuals with a lower RNFL thickness were more likely to have type 2 diabetes (lowest vs highest tertile of thickness, 352 patients [24.9%] vs 261 patients [18.4%]), had higher systolic blood pressure (mean [SD], 134.8 [17.3] mm Hg for lowest tertile of thickness vs 131.9 [18.0] mm Hg for highest tertile of thickness), and were more likely to use antihypertensive medication (lowest vs highest tertile of thickness, 570 patients [40.3%] vs 449 patients [31.7%]). The median (IQR) follow-up time was 5.0 (3.0-6.0) years, and there were 445 participants with incident clinically relevant depressive symptoms. General characteristics of the participants with and without missing data were highly comparable (eTable 2 in the Supplement).

**Table 1.** Baseline Characteristics of the Prospective Study Population for Incidence of Clinically Relevant Depressive Symptoms According to Tertiles of RNFL Thickness

Characteristic	Participants, No. (%)			
	Study population (N = 4247)	RNFL thickness		
		Tertile 1, low (n = 1415)	Tertile 2, middle (n = 1416)	Tertile 3, high (n = 1416)
Demographic and clinical variables				
Age, mean (SD), y	59.7 (8.4)	60.3 (8.3)	59.5 (8.4)	59.3 (8.5)
Sex				
Female	2159 (50.8)	616 (43.5)	752 (53.1)	791 (55.9)
Male	2088 (49.2)	799 (56.5)	664 (46.9)	625 (44.1)
Glucose metabolism status				
Type 2 diabetes	870 (20.5)	352 (24.9)	257 (18.1)	261 (18.4)
Other type of diabetes	18 (0.4)	8 (0.6)	5 (0.4)	5 (0.4)
Prediabetes	649 (15.3)	218 (15.4)	219 (15.5)	212 (15.0)
Normal glucose metabolism	2710 (63.8)	837 (59.2)	935 (66.0)	938 (66.2)
Educational status				
Low	1379 (32.5)	421 (29.8)	480 (33.9)	478 (33.8)
Middle	1187 (27.9)	371 (26.2)	390 (27.5)	426 (30.1)
High	1681 (39.6)	623 (44.0)	546 (38.6)	512 (36.2)
Waist circumference, mean (SD), cm	94.0 (13.0)	95.3 (13.1)	93.2 (12.7)	93.5 (13.2)
Ratio of total cholesterol to high-density lipoprotein cholesterol, median (IQR)	3.33 (2.8-4.1)	3.36 (2.8-4.1)	3.33 (2.8-4.1)	3.3 (2.7-4.1)
Use of lipid-modifying medication	1246 (29.3)	454 (32.1)	391 (27.6)	401 (28.3)
Office blood pressure, mean (SD), mm Hg				
Systolic	133.2 (17.8)	134.8 (17.3)	132.8 (17.9)	131.9 (18.0)
Diastolic^a^	75.5 (9.7)	76.5 (9.7)	75.2 (9.7)	74.8 (9.7)
Use of antihypertensive medication	1482 (34.9)	570 (40.3)	463 (32.7)	449 (31.7)
Smoking status				
Nonsmoker	1651 (38.9)	558 (39.4)	523 (36.9)	570 (40.3)
Former	2130 (50.2)	721 (51.0)	739 (52.2)	670 (47.3)
Current	466 (11.0)	136 (9.6)	154 (10.9)	176 (12.4)
Alcohol consumption				
None	682 (16.1)	204 (14.4)	234 (16.5)	244 (17.2)
Low	2507 (59.0)	836 (59.1)	814 (57.5)	857 (60.5)
High	1058 (24.9)	375 (26.5)	368 (26.0)	315 (22.2)
Partner status (with partner)	3510 (82.6)	1170 (82.7)	1155 (81.6)	1185 (83.7)
Peripapillary RNFL thickness, mean (SD), μm	94.8 (10.9)	83.3 (6.3)	95.2 (2.5)	106.0 (6.6)
Depressive symptoms				
Use of antidepressive medication	235 (5.5)	79 (5.6)	85 (6.0)	71 (5.0)
Major depressive disorder at baseline^b^	144 (3.0)	54 (3.4)	40 (2.5)	50 (3.1)
Clinically relevant depressive symptoms at baseline^c^	236 (4.6)	69 (4.0)	86 (5.0)	81 (4.7)
Patient Health Questionnaire–9 score, median (IQR)				
Baseline^c^	2 (0-4)	2 (0-4)	2 (0-4)	2 (0-4)
Years after baseline^d^				
1	2 (0-4)	2 (0-4)	2 (0-4)	2 (0-4)
2	2 (0-4)	2 (0-4)	2 (0-4)	2 (0-4)
3	2 (0-4)	2 (0-4)	2 (0-4)	2 (0-4)
4	2 (0-4)	2 (0-4)	2 (0-4)	2 (0-4)
5	2 (0-4)	2 (0-4)	2 (0-4)	2 (0-4)
6	2 (0-4)	2 (0-4)	2 (0-4)	2 (0-4)
7	2 (0-4)	2 (0-4)	2 (0-4)	2 (0-4)
Incident clinically relevant depressive symptoms	445 (10.5)	162 (11.4)	150 (10.6)	133 (9.4)

Abbreviation: RNFL, retinal nerve fiber layer thickness.

^a^ Data for office diastolic blood pressure were available for 4246 participants.

^b^ Data are presented for the study population at baseline with complete data on the presence of a major depressive disorder, 5071 participants.

^c^ Data are presented for the study population at baseline with complete data on (clinically relevant) depressive symptoms, 5170 participants.

^d^ Data are presented for the prospective study population with complete data on depressive symptoms per moment of assessment (4934 participants; the number of with data available per year is reported in the legend of Figure 1). At all follow-up moments the median Patient Health Questionnaire–9 score and IQR values in the general population were numerically identical.

### Main Analyses

**Figure 2.**
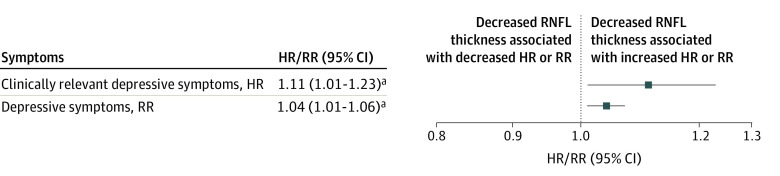
Associations of Lower Retinal Nerve Fiber Layer (RNFL) Thickness (per SD) With Incidence of Clinically Relevant Depressive Symptoms and Depressive Symptoms Over Time A higher hazard ratio (HR) or rate ratio (RR) indicates higher incidence of clinically relevant depressive symptoms or more depressive symptoms over time. One SD of RNFL thickness corresponds to 10.9 µm in the incidence of clinically relevant depressive symptoms study population and 11.0 µm in the depressive symptoms over time study population. Associations are adjusted for age, sex, glucose metabolism status, and education status, waist circumference, total cholesterol to high-density lipoprotein cholesterol ratio, use of lipid-modifying medication, office systolic blood pressure, use of antihypertensive medication, smoking, alcohol consumption, and partner status. ^a^*P* < .05.

**Figure 3.**
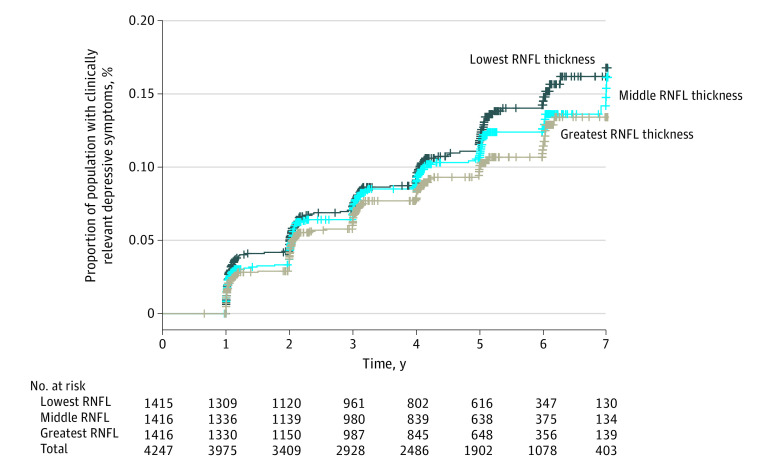
Kaplan-Meier Plot for Hazard of Incidence of Clinically Relevant Depressive Symptoms Black line indicates hazard of incidence of clinically relevant depressive symptoms for participants with retinal nerve fiber layer (RNFL) thickness less than 90.75 µm (ie, lowest RNFL thickness, indicating greatest extent of neurodegeneration). Blue line indicates hazard of incidence of clinically relevant depressive symptoms for participants with RNFL thickness 90.76 to 99.59 µm. Gray line indicates hazard of incidence of clinically relevant depressive symptoms for participants with RNFL thickness greater than or equal to 99.60 µm (ie, greatest RNFL thickness, indicating lowest extent of neurodegeneration). Of the participants with prospective data on depressive symptoms, 4822, 4280, 3911, 3467, 2854, 1636, and 710 participants had data on depressive symptoms after 1, 2, 3, 4, 5, 6, or 7 years, respectively, after baseline.

After full adjustment (model 2), lower RNFL thickness was significantly associated with a higher incidence of clinically relevant depressive symptoms (PHQ-9 score ≥10 points) and more depressive symptoms over time (per 1 SD, hazard ratio, 1.11 [95% CI, 1.01-1.23]; rate ratio, 1.04 [95% CI, 1.01-1.06]) (Figure 2 and Table 2). Figure 3 shows the Kaplan-Meier curve. Time, sex, and glucose metabolism status did not modify any of the associations (*P* values for interaction are presented in eTable 3 in the Supplement).

**Table 2.** Associations of Lower RNFL Thickness (per SD) With Incidence of Clinically Relevant Depressive Symptoms and Depressive Symptoms Over Time

Regression coefficient^a^	Crude	Model 1^b^	Model 2^c^
Incidence of clinically relevant depressive symptoms, HR (95% CI)	1.08 (0.99-1.19)	1.11 (1.01-1.22)^d^	1.11 (1.01-1.23)^d^
Depressive symptoms over time, RR (95% CI)	1.02 (1.00-1.05)	1.04 (1.01-1.06)^d^	1.04 (1.01-1.06)^d^

Abbreviations: HR, hazard ratio; RNFL, retinal nerve fiber layer thickness; RR, rate ratio.

^a^ Regression coefficients represent the HR for the incidence of clinically relevant depressive symptoms or RR for depressive symptoms over time per SD lower RNFL thickness, where a higher HR or RR indicates higher incidence of clinically relevant depressive symptoms or more depressive symptoms over time. One SD of RNFL thickness corresponds with 10.9 µm in the incidence of clinically relevant depressive symptoms study population and 11.0 µm in the depressive symptoms over time study population.

^b^ Model 1 includes age, sex, glucose metabolism status, and educational status.

^c^ Model 2 includes model 1 plus waist circumference, total cholesterol to high-density lipoprotein cholesterol ratio, use of lipid-modifying medication, office systolic blood pressure, use of antihypertensive medication, smoking, alcohol consumption, and partner status.

^d^ *P* < .05.

### Additional Analyses

We generally had results numerically similar to those shown in Table 2 in additional analyses (shown in eTable 4, eTable 5, eTable 6, and eTable 7 in the Supplement); that is, the results did not materially change when we either adjusted for covariates not included in the main analyses, when we excluded certain individuals from analyses, or when we replaced certain covariates with similar covariates (all results are presented in detail in eAppendix 2 in the Supplement). However, only when educational status was replaced with income level or occupational level was the strength of the associations under study attenuated (eTable 7 in the Supplement).

## Discussion

The present population-based cohort study found that lower RNFL thickness was significantly associated with a higher incidence of clinically relevant depressive symptoms and more depressive symptoms over time, after adjustment for demographic, cardiovascular, and lifestyle factors. To our knowledge, this study is the first to investigate the association between RNFL thickness and depression as well as the first population-based study to investigate the association between RNFL thickness and depression. Our results are consistent with the hypothesis that neurodegeneration is associated with the early pathobiology of late-life depression. Lower RNFL thickness is thought to reflect more retinal neurodegeneration, whereas more symptoms of late-life depression are thought to reflect more cerebral neurodegeneration in brain areas involved in mood, cognition, and motor behavior, such as the hippocampus and the frontal-subcortical brain areas.^1,5^

Mechanistically, causes of neurodegeneration are thought to be chronic stress, microvascular dysfunction, amyloid accumulation, and adverse exposure to risk factors such as hyperglycemia. First, chronic exposure to stress is thought to lead to elevated levels of cortisol, which may increase levels of glucocorticoids and induce neurodegeneration.^1,4^ Second, both microvascular dysfunction and amyloid accumulation are thought to contribute to impaired functioning of hemodynamic autoregulation, which can predispose to ischemia and lead to higher levels of neuroinflammation, which is detrimental for neuronal cells.^1,2,8^ Indeed, retinal and brain neuronal cells are highly susceptible to ischemia because they have a high energy demand and are fully dependent on the continuous supply of nutrients via the microvasculature.^35^ Third, amyloid accumulation and hyperglycemia are thought to be neurotoxic and can lead to neurodegeneration.^36^

Associations were attenuated when educational status was replaced with income level or occupational level, possibly because such adjustments may lead to collider bias. Neurodegeneration and depression may lead to lower income and occupation level, and, therefore, adjustment for these covariates may attenuate the strength of the associations under study.^37,38^ Methodologically, conditioning on a common outcome may induce a spurious estimate of the association between the independent and dependent variable.^37,38^

Our findings may be clinically relevant. First, RNFL thickness may be a feasible biomarker for identification of individuals at risk for late-life depression as its measurement is noninvasive and inexpensive.^39^ Indeed, RNFL thickness has been found to be a promising early biomarker for other neurodegenerative diseases such as multiple sclerosis.^40^ Second, at an early stage there may be an opportunity to prevent depression by targeting early neurodegeneration via reducing exposure to detrimental risk factors, such as hyperglycemia, and lifestyle factors, such as an unhealthy diet.^41,42^ Further investigation is warranted to investigate whether the early-stage modification of cardiovascular risk factors and adverse lifestyle factors may relevantly prevent neurodegeneration and, via neurodegeneration, the risk of incident clinically relevant depressive symptoms.^41,42^

### Strengths and Limitations

This study has the following strengths. First, the use of data from a large population-based cohort study reduces the chance that our results are affected by selection bias and enables us to draw conclusions that are valid in the general population.^43^ Second, because of the prospective nature of the data we could account for temporality and, thus, can conclude that lower RNFL thickness precedes incident clinically relevant depressive symptoms.^44^ Third, we adjusted for a large number of potential confounders, which reduces the chance that unmeasured confounding spuriously affects the strength of associations under study (ie, confounding bias).^43^ Fourth, all variables included in this study were assessed in a standardized manner with state-of-the-art methods (eg, RNFL thickness), which reduces the chance that measurement error affects associations under study (ie, information bias).^43^

This study also has limitations. First, the presence of a clinically relevant depressive disorder was prospectively assessed with the PHQ-9, which is a reasonably valid instrument for the diagnosis of the presence of a major depressive disorder (ie, sensitivity 88% and specificity 85% at a cutoff score of ≥10),^45^ but is not the current reference standard used in the clinic.^14^ Second, we may have underestimated the strength of the association between RNFL thickness and late-life depression if participants with depressive symptoms or a major depressive disorder are more likely to have missing data.^43^ For example, participants may be less likely to take part in a study or complete a yearly questionnaire during a depressive episode. Third, we may have underestimated the strength of the association between RNFL thickness and late-life depression because we could not account for the use of antidepressive medication that started after the baseline measurement. Fourth, even though we took an extensive set of confounders into account, we cannot fully exclude unmeasured confounding.^46^ For example, exposure to confounders may change over time, and in this study confounders were assessed at baseline only. Another example is that, in additional analyses, we adjusted for the presence, but not the severity, of glaucoma. Fifth, some measurement error may have occurred as depressive symptoms were measured at only 1 time point per year (ie, data are interval-censored) and the amount and intensity of depressive symptoms can fluctuate over time because of the nature of depression.^47^ This may have led to an underestimation of the strength of prospective associations under study via regression dilution bias.^48^ Sixth, we studied White individuals aged 40 to 75 years who volunteered to take part in a population-based study, and, therefore, our results may be generalizable to such a population; whether these results also apply to other populations requires further study.

## Conclusions

In summary, the present population-based cohort study found that lower RNFL thickness was associated with a higher incidence of clinically relevant depressive symptoms and more depressive symptoms over time. Hence, neurodegeneration may contribute to the early pathobiology of late-life depression and monitoring of retinal neurodegeneration may provide a means to identify individuals at risk for late-life depression.

## Notes

Supplement. **eAppendix 1.** Supplemental Methods **eAppendix 2.** Supplemental Results
eReferences
**eFigure 1.** Examples of Quality of Centering of Circular Scans on the Optic Nerve Head **eFigure 2.** Examples of Poor Imaging Quality and Scan Errors **eTable 1.** Additional General Characteristics of the Prospective Study Population With Complete Data on the Incidence of Clinically Relevant Depressive Symptoms, According to Tertiles of RNFL Thickness **eTable 2.** General Characteristics of the Participants With Complete Data and Missing Data in the Prospective Study Population With Complete Data on the Incidence of Clinically Relevant Depressive Symptoms **eTable 3.** *P* values of Interaction Terms of RNFL Thickness With Sex, Glucose Metabolism Status, or Time **eTable 4.** Cross-sectional Associations of Lower RNFL Thickness (per SD) With (Clinically Relevant) Depressive Symptoms or the Presence of a Major Depressive Disorder **eTable 5.** Associations of Lower RNFL Thickness (per SD) With the Incidence of Clinically Relevant Depressive Symptoms and Depressive Symptoms Over Time, Additional Analyses **eTable 6. **Associations of Lower RNFL Thickness (per SD) With the Incidence of Clinically Relevant Depressive Symptoms and Depressive Symptoms Over Time, Additionally Adjusted for Physical Activity (Model 3), Diet Score (Model 4), Spherical Equivalent (Model 5), Use of Antidepressive Medication (Model 6), Kidney Variables (Model 7), Prior Cardiovascular Disease (Model 8), Retinopathy (Model 9), Glaucoma (Model 10), and Plasma Biomarkers of Low-grade Inflammation (Model 11), or After Exclusion of Individuals With Retinopathy (Model 12), or Glaucoma (Model 13) **eTable 7.** Associations of Lower RNFL Thickness (per SD) With the Incidence of Clinically Relevant Depressive Symptoms and Depressive Symptoms Over Time, Replacement of Glucose Metabolism Status With Fasting Plasma Glucose (Model 3A), 2-hour Post Load Glucose (Model 3B) or HbA1c (Model 3C), Waist Circumference With BMI (Model 3D), Office Systolic Blood Pressure With Office Diastolic Blood Pressure (Model 3E), 24-hour Ambulatory Systolic Blood Pressure (Model 3F) and 24-hour Ambulatory Diastolic Blood Pressure (Model 3G), and Educational Status With Income Level (Model 3H) or Occupational Status (Model 3I)
