# Supplementary material for: Association of Retinal Nerve Fiber Layer Thickness, an Index of Neurodegeneration, With Depressive Symptoms Over Time
Source: JAMA Netw Open. 2021 Nov 16;4(11):e2134753. doi: 10.1001/jamanetworkopen.2021.34753 (PMC8596200; doi:10.1001/jamanetworkopen.2021.34753)
Supplement: Supplement. — eAppendix 1. Supplemental Methods eAppendix 2. Supplemental Results eReferences eFigure 1. Examples of Quality of Centering of Circular Scans on the Optic Nerve Head eFigure 2. Examples of Poor Imaging Quality and Scan Errors eTable 1. Additional General Characteristics of the Prospective Study Population With Complete Data on the Incidence of Clinically Relevant Depressive Symptoms, According to Tertiles of RNFL Thickness eTable 2. General Characteristics of the Participants With Complete Data and Missing Data in the Prospective Study Population With Complete Data on the Incidence of Clinically Relevant Depressive Symptoms eTable 3. P values of Interaction Terms of RNFL Thickness With Sex, Glucose Metabolism Status, or Time eTable 4. Cross-sectional Associations of Lower RNFL Thickness (per SD) With (Clinically Relevant) Depressive Symptoms or the Presence of a Major Depressive Disorder eTable 5. Associations of Lower RNFL Thickness (per SD) With the Incidence of Clinically Relevant Depressive Symptoms and Depressive Symptoms Over Time, Additional Analyses eTable 6. Associations of Lower RNFL Thickness (per SD) With the Incidence of Clinically Relevant Depressive Symptoms and Depressive Symptoms Over Time, Additionally Adjusted for Physical Activity (Model 3), Diet Score (Model 4), Spherical Equivalent (Model 5), Use of Antidepressive Medication (Model 6), Kidney Variables (Model 7), Prior Cardiovascular Disease (Model 8), Retinopathy (Model 9), Glaucoma (Model 10), and Plasma Biomarkers of Low-grade Inflammation (Model 11), or After Exclusion of Individuals With Retinopathy (Model 12), or Glaucoma (Model 13) eTable 7. Associations of Lower RNFL Thickness (per SD) With the Incidence of Clinically Relevant Depressive Symptoms and Depressive Symptoms Over Time, Replacement of Glucose Metabolism Status With Fasting Plasma Glucose (Model 3A), 2-hour Post Load Glucose (Model 3B) or HbA1c (Model 3C), Waist Circumference With BMI (Model 3D), Office Systolic Blood Pressure [file jamanetwopen-e2134753-s001.pdf]

Supplemental Online Content

van der Heide FCT, Steens ILM, Geraets AFJ, et al. Association of retinal nerve fiber layer thickness, an index of neurodegeneration, with depressive symptoms over time. *JAMA Netw Open*. 2021;4(11):e2134753.
doi:10.1001/jamanetworkopen.2021.34753

eAppendix 1. Supplemental Methods
eAppendix 2. Supplemental Results
eReferences
eFigure 1. Examples of Quality of Centering of Circular Scans on the Optic Nerve Head
eFigure 2. Examples of Poor Imaging Quality and Scan Errors
eTable 1. Additional General Characteristics of the Prospective Study Population With Complete Data on the Incidence of Clinically Relevant Depressive Symptoms, According to Tertiles of RNFL Thickness
eTable 2. General Characteristics of the Participants With Complete Data and Missing Data in the Prospective Study Population With Complete Data on the Incidence of Clinically Relevant Depressive Symptoms
eTable 3. *P* values of Interaction Terms of RNFL Thickness With Sex, Glucose Metabolism Status, or Time
eTable 4. Cross-sectional Associations of Lower RNFL Thickness (per SD) With (Clinically Relevant) Depressive Symptoms or the Presence of a Major Depressive Disorder
eTable 5. Associations of Lower RNFL Thickness (per SD) With the Incidence of Clinically Relevant Depressive Symptoms and Depressive Symptoms Over Time, Additional Analyses
eTable 6. Associations of Lower RNFL Thickness (per SD) With the Incidence of Clinically Relevant Depressive Symptoms and Depressive Symptoms Over Time, Additionally Adjusted for Physical Activity (Model 3), Diet Score (Model 4), Spherical Equivalent (Model 5), Use of Antidepressive Medication (Model 6), Kidney Variables (Model 7), Prior Cardiovascular Disease (Model 8), Retinopathy (Model 9), Glaucoma (Model 10), and Plasma Biomarkers of Low-grade Inflammation (Model 11), or After Exclusion of Individuals With Retinopathy (Model 12), or Glaucoma (Model 13)
eTable 7. Associations of Lower RNFL Thickness (per SD) With the Incidence of Clinically Relevant Depressive Symptoms and Depressive Symptoms Over Time, Replacement of Glucose Metabolism Status With Fasting Plasma Glucose (Model 3A), 2-hour Post Load Glucose (Model 3B) or Hb_{A1c} (Model 3C), Waist Circumference With BMI (Model 3D), Office Systolic Blood Pressure With Office Diastolic Blood Pressure (Model 3E), 24-hour Ambulatory Systolic Blood Pressure (Model 3F) and 24-hour Ambulatory Diastolic Blood Pressure (Model 3G), and Educational Status With Income Level (Model 3H) or Occupational Status (Model 3I)

This supplemental material has been provided by the authors to give readers additional information about their work.

© 2021 van der Heide FCT et al. *JAMA Network Open.*

eAppendix 1. Supplemental Methods

Depressive symptoms

Presence and severity of clinically relevant depressive symptoms were assessed with a validated Dutch version of the Patient Health Questionaire-9 (PHQ-9) at baseline and during seven years of annual follow-up.[1] The PHQ-9 is a self-administered questionnaire based on the Diagnostic and Statistical Manual of Mental Disorders, fourth edition (DSM-IV) criteria for a major depressive disorder and consists of nine items.[2] We used PHQ-9 measurements with complete data, except that, in case when one or two items were missing, we recalculated the total score as 9 × (total points/9 minus number of missing items) and rounded to the nearest integer. Presence of clinically relevant depressive symptoms was defined as PHQ-9 score ≥10. [3] Incident clinically relevant depressive symptoms was defined as the absence of clinically relevant depressive symptoms at baseline (PHQ-9 score <10) and the presence of clinically relevant depressive symptoms (PHQ-9 score ≥10) at (at least) one follow-up assessment.

Major depressive disorder

The presence of a major depressive disorder in the preceding two weeks was assessed with the Mini-International Neuropsychiatric Interview (MINI), a short diagnostic structured interview, according to the DSM-IV criteria. [4] Major depressive disorder was defined as the presence of at least one core symptom (i.e. depressed mood or loss of interest) and at least 4 other symptoms of depression (i.e. another core symptom, significant weight change or change in appetite, insomnia or hypersomnia, psychomotor agitation or retardation, fatigue or loss of energy, guilt or worthlessness, diminished ability to think or concentrate or indecisiveness, and suicidal thoughts or plans). Then, lifetime history of a major depressive disorder was assessed with the MINI and defined as the presence of symptoms of a major depressive disorder for at minimum two weeks during lifetime. Age of onset of lifetime major depressive disorder was defined as the youngest age at which a major depressive disorder occurred based on lifetime history. Presence of a major depressive disorder and lifetime history of a depressive disorder were both assessed at baseline.

Assessment of the retinal nerve fiber layer thickness

All participants were asked to refrain from smoking and drinking caffeine-containing beverages three hours before the measurement. A light meal (breakfast or lunch), low in fat content, was allowed if taken at least 90 minutes prior to the start of the measurements. Pupils were dilated with topical 0.5% tropicamide and 2.5% phenylephrine at least 15 minutes prior to the start of the examination.

We assessed RNFL thickness with optical coherence tomography (OCT; Spectralis unit and Eye Explorer version 5.7.5.0 software; Heidelberg Engineering, Heidelberg, Germany). The RNFL thickness (μm) of both eyes was measured within a 3.45 mm diameter circular scan (12°, 768 voxels, 100 automatic real-time tracking) centred on the optic nerve head by trained research assistants according to a standard operating procedure. The peripapillary OCT scans were reviewed and scored for the presence of measurement errors by experienced graders based on a predefined protocol. Graders were masked to clinical information of the participants. OCT images were excluded if one of the following criteria was present: scan errors (i.e. incomplete scan, poor centering of the circular scan

© 2021 van der Heide FCT et al. *JAMA Network Open.*

on the optic nerve head, RNFL layer incorrectly defined, or technical problem with the OCT device) and/or poor imaging quality (signal-to-noise ratio<15 dB). To reduce measurement error, the average RNFL thickness of both eyes was used in analyses (50.0% of participants). If data were only available for one eye the RNFL thickness was defined as the RNFL thickness of the eye for which data were available (50% of participants). The intra- and interrater reliability for the assessment of RNFL thickness are 0.966 and 0.963, respectively.[5]

Grading of OCT circle scans

OCT scans were considered of sufficient quality if all the following criteria were met: good centering of the circular scan on the optic nerve head (examples of good, poor and very poor centering are shown in Supplemental eFigure S1); complete (data of all 768 voxels was available); automatic quality ≥15 dB (an example of a scan with poor quality imaging is shown in Supplemental eFigure S2); no measurement error present (examples of all assessed measurement errors are shown in Supplemental eFigure S2). The percentage of agreement for selection of scans with sufficient quality ranged between 90% and 94% for four trained graders and was 70% for one grader (n=50 OCT scans per comparison).

Additional covariates

As previously described, we assessed history of cardiovascular disease by questionnaire, height (cm) and weight (kg) during a physical examination, and hemoglobin A1c (HbA1c) (mmol/mol), plasma biomarkers of low-grade inflammation (high sensitivity C-reactive protein (CRP) (µg/ml), serum amyloid A (SAA) (µg/ml), interleukin-6 (IL-6) (pg/ml), interleukin-8 (IL-8) (pg/ml) and tumour necrosis factor alpha (TNF-α) (pg/ml)) in fasting blood samples; 24-hour urinary albumin excretion (twice), defined as normal (<15 mg/24h), micro- (15-<30 mg/24h) and macroalbuminuria (≥30 mg/24h); estimated glomerular filtration rate (eGFR; in ml/min/1.73 m2), calculated the estimated glomerular filtration rate (eGFR) based on serum creatinine only, since cystatin C was not presently available in all study participants; physical activity by accelerometer;[6] dietary intake with a validated food-frequency questionnaire (i.e. the Dutch Healthy Diet score);[7] spherical equivalent and intraocular pressure in both eyes with an automated refractor (Tonoref II; Nidek, Gamagori, Japan);[8, 9] presence of retinopathy on fundus photos;[8] and 24-hour systolic and diastolic ambulatory blood pressure by use of an automated oscillometric upper arm blood pressure monitor (Omron 705IT, Omron, Japan).[9] The Dutch Healthy Diet score was recalculated so that the ‘adjusted dietary intake score’ reflects dietary intake without alcohol consumption (so that alcohol consumption and dietary intake could separately be included in statistical models). Body mass index (BMI) was calculated as kilograms per square meters (BMI=weight/length^2). Glaucoma was defined as use of intraocular pressure-lowering medication or an intraocular pressure higher than 21 mm Hg in any eye (91.3% of all participants had data on intraocular pressure available for at least 1 eye). Spherical equivalent was defined as the mean spherical equivalent of both eyes (available for 91.1% of all participants) or as the spherical equivalent of the eye for which data was available. Use of anti-depressive medication was defined as use of one of the following types of medication: (selective or non-selective) serotonin reuptake inhibitor, tricyclic antidepressant, tetracyclic antidepressant, monoamine oxidase inhibitor, melatonin receptor agonist, or atypical antidepressant. Last, educational level was assessed via a questionnaire, where education was categorized into eight categories of education, i.e. 1. no education, 2. primary education, 3. lower vocational education, 4. general secondary education, 5. general vocational education, 6. higher secondary and pre-

© 2021 van der Heide FCT et al. *JAMA Network Open.*

university education, 7. higher vocational education and 8. university. We composed three categories of educational level, i.e. low (1–3), middle (4–6), and high (7 and 8), as previously described.[10]

Statistical analysis

We used negative binomial regression analyses to investigate the cross-sectional association between standardized RNFL thickness and depressive symptoms. We used negative binomial regression to account for the right-skewed PHQ-9 score which contained many null values. Results were expressed as rate ratio (RR) with corresponding 95% confidence interval (95% CI). Then, we used multivariable logistic regression analyses to cross-sectionally investigate the association between standardized RNFL thickness and the presence of clinically relevant depressive symptoms and the association between standardized RNFL thickness and the presence of a major depressive disorder. Results were expressed as odds ratio (OR) with corresponding 95% CI.

We used collinearity diagnostics (i.e. tolerance <0.10 and/or variance inflation factor >10) to detect multicollinearity between covariates (collinearity diagnostics were only available in cross-sectional analyses).

Additional analyses

We performed a number of additional analyses which are not shown in the Main Methods section. Here we provide an overview of all additional analyses we performed. First, we investigated the cross-sectional association between RNFL thickness and the presence of a major depressive disorder (assessed with the Mini-International Neuropsychiatric Interview, the gold standard for the assessment of a major depressive disorder).[4] Second, we studied the cross-sectional associations of RNFL thickness with depressive symptoms (continuous) and with the presence of clinically relevant depressive symptoms (present/absent). Third, we performed additional prospective analyses in which we excluded participants with use of anti-depressive medication at baseline. Fourth, we repeated prospective analyses where we excluded participants with a major depressive disorder at baseline in addition to participants with clinically relevant depressive symptoms at baseline. Fifth, we studied prospective associations without exclusion of participants with clinically relevant depressive symptoms at baseline.[11] Sixth, we conducted prospective analyses in which we excluded participants without incident clinically relevant depressive symptoms when they had more than 2 missing assessments of PHQ-9 data during follow-up. Seventh, we studied associations after exclusion of participants with an age of onset of major depressive disorder ≤ 40 years. We excluded participants with an age of onset of major depressive disorder ≤ 40 years because the pathobiology of depression in these participants (i.e. early-life depression) may differ from the pathobiology of depression in participants with older age of onset of depression (i.e. late-life depression).[12] Eighth, we additionally adjusted for accelerometer-assessed physical activity,[13] diet score,[13] and spherical equivalent.[14]Adjustment for these potential confounders was not included in the main analyses because data were missing for a relatively large number of participants (up to N=617 participants had missing data on one or more of these variables). Nineth, we additionally adjusted for use of anti-depressive medication (assessed at baseline). As anti-depressive medication use may be a confounder and/or descendent of the outcome we adjusted for these covariates in additional analyses.[15] Tenth, we additionally adjusted for kidney variables (estimated glomerular filtration rate and albuminuria),[16] prior cardiovascular disease,[17] retinopathy,[18] glaucoma,[19] and plasma biomarkers of low-grade inflammation[20] (all assessed at baseline) as they may be confounders and/or also (in part) be potential mediators. Eleventh, we performed additional

analyses in which we excluded participants with baseline retinopathy or glaucoma. Last, we replaced glucose metabolism status with fasting plasma glucose, 2-hour post load plasma glucose, or hemoglobin A1c (HbA1c); waist circumference with body mass index; office systolic blood pressure with office diastolic blood pressure, or systolic or diastolic 24-hour ambulatory blood pressure; and educational status with income level or occupational status.

eAppendix 2. Supplemental Results

Generally, we observed quantitively similar results in a range of additional analyses. First, in cross-sectional analyses, lower RNFL thickness was statistically significantly associated with more depressive symptoms but not with clinically relevant depressive symptoms, or the presence of a major depressive disorder (Supplemental eTable S4). Second, associations did not materially change when we excluded participants with use of anti-depressive medication at baseline, when we excluded participants with a major depressive disorder at baseline in addition to participants with clinically relevant depressive symptoms at baseline, or when we studied associations without exclusion of participants with clinically relevant depressive symptoms at baseline (Supplemental eTable S5). Third, we found numerically comparable associations when we excluded participants without incident clinically relevant depressive symptoms when they had more than 2 missing assessments of PHQ-9 data during follow-up and when we excluded participants with an age of onset of major depressive disorder ≤ 40 years (Supplemental eTable S5). Fourth, associations remained similar when we additionally adjusted for physical activity, diet score, or spherical equivalent, use of anti-depressive medication, kidney variables, prior cardiovascular disease, retinopathy, glaucoma, or plasma biomarkers of low-grade inflammation (Supplemental eTable S6). Fifth, associations were similar when we excluded participants with retinopathy or glaucoma (Supplemental eTable S6). Sixth, associations remained similar after substitution of glucose metabolism by fasting plasma glucose, 2-hour post load plasma glucose, or HbA1c; waist circumference by body mass index; and office systolic blood pressure by office diastolic blood pressure, or 24-hour ambulatory mean systolic or diastolic blood pressure (Supplemental eTable S7). However, associations were less strong after substitution of educational status by income level or occupational status (Supplemental eTable S7).

© 2021 van der Heide FCT et al. *JAMA Network Open.*

eReferences

1. Kroenke K, Spitzer R and Williams J. The PHQ-9: Validity of a brief depression severity measure. *Gen Intern Med*. 2001;16:606-13.
2. Bell CC. DSM-IV: Diagnostic and Statistical Manual of Mental Disorders. . 1994;272(10):828.
3. Pettersson A, Bostrom KB, Gustavsson P and Ekselius L. Which instruments to support diagnosis of depression have sufficient accuracy? A systematic review. *Nordic journal of psychiatry*. 2015;69:497-508.
4. DVSheehan Y, KHSheehan P, JJanavs E and THergueta R. GCDunbar (1998). The Mini-International Neuropsychiatric Interview (MINI): the development and validation of a structured diagnostic psychiatric interview for DSM-IV and ICD-10. *Journal of Clinical Psychiatry*. 59:22-33.
5. Hong JT, Sung KR, Cho JW, Yun SC, Kang SY and Kook MS. Retinal nerve fiber layer measurement variability with spectral domain optical coherence tomography. *Korean J Ophthalmol*. 2012;26:32-8.
6. Sorensen BM, van der Heide FCT, Houben A, Koster A, T TJMB, J SAGS, Kroon AA, van der Kallen CJH, Henry RMA, van Dongen M, S JPME, H HCMS, van der Berg JD, Schaper NC, Schram MT and Stehouwer CDA. Higher levels of daily physical activity are associated with better skin microvascular function in type 2 diabetes-The Maastricht Study. *Microcirculation*. 2020;27:e12611.
7. Looman M, Feskens EJM, de Rijk M, Meijboom S, Biesbroek S, Temme EHM, de Vries J and Geelen A. Development and evaluation of the Dutch Healthy Diet index 2015. *Public Health Nutr*. 2017;20:2289-2299.
8. De Clerck EEB, Schouten JSAG, Berendschot TTJM, Goezinne F, Dagnelie PC, Schaper NC, Schram MT, Stehouwer CDA and Webers CAB. Macular thinning in prediabetes or type 2 diabetes without diabetic retinopathy: the Maastricht Study. *Acta Ophthalmol*. 2018;96:174-182.
9. Schram MT, Sep SJ, van der Kallen CJ, Dagnelie PC, Koster A, Schaper N, Henry RM and Stehouwer CD. The Maastricht Study: an extensive phenotyping study on determinants of type 2 diabetes, its complications and its comorbidities. *Eur J Epidemiol*. 2014;29:439-51.
10. Qi Y, Koster A, van Boxtel M, Kohler S, Schram M, Schaper N, Stehouwer C and Bosma H. Adulthood Socioeconomic Position and Type 2 Diabetes Mellitus-A Comparison of Education, Occupation, Income, and Material Deprivation: The Maastricht Study. *Int J Environ Res Public Health*. 2019;16.
11. Tizabi Y. Duality of Antidepressants and Neuroprotectants. *Neurotoxicity Research*. 2016;30:1-13.
12. Gollan J, Raffety B, Gortner E and Dobson K. Course profiles of early- and adult-onset depression. *J Affect Disord*. 2005;86:81-6.
13. Farioli Vecchioli S, Sacchetti S, Nicolis di Robilant V and Cutuli D. The Role of Physical Exercise and Omega-3 Fatty Acids in Depressive Illness in the Elderly. *Curr Neuropharmacol*. 2018;16:308-326.
14. Bikbov MM, Kazakbaeva GM, Gilmanshin TR, Zainullin RM, Arslangareeva, II, Salavatova VF, Bikbova GM, Panda-Jonas S, Nikitin NA, Zaynetdinov AF, Nuriev IF, Khikmatullin RI, Uzianbaeva YV, Yakupova DF, Aminev SK and Jonas JB. Axial length and its associations in a Russian population: The Ural Eye and Medical Study. *PLoS One*. 2019;14:e0211186.
15. Bubl E, Ebert D, Kern E, van Elst LT and Bach M. Effect of antidepressive therapy on retinal contrast processing in depressive disorder. *Br J Psychiatry*. 2012;201:151-8.

© 2021 van der Heide FCT et al. *JAMA Network Open.*

16. Hedayati SS, Jiang W, O'Connor CM, Kuchibhatla M, Krishnan KR, Cuffe MS, Blazing MA and Szczech LA. The association between depression and chronic kidney disease and mortality among patients hospitalized with congestive heart failure. *Am J Kidney Dis*. 2004;44:207-215.
17. Hare DL, Toukhsati SR, Johansson P and Jaarsma T. Depression and cardiovascular disease: a clinical review. *Eur Heart J*. 2014;35:1365-U13.
18. Chen X, Nie C, Gong Y, Zhang Y, Jin X, Wei S and Zhang M. Peripapillary retinal nerve fiber layer changes in preclinical diabetic retinopathy: a meta-analysis. *PLoS One*. 2015;10:e0125919.
19. Satyaprakash S VS, Arun Kumar S, Shivangi S, Kamaljeet S, Jagriti R. A Comparison of Retinal Nerve Fibre Layer Thickness by Spectral Domain Optical Coherence Tomography in Primary Open Angle Glaucoma, Normotensive Glaucoma and Glaucoma Suspect. 2018;3.
20. van Dooren FE, Schram MT, Schalkwijk CG, Stehouwer CD, Henry RM, Dagnelie PC, Schaper NC, van der Kallen CJ, Koster A and Sep SJ. Associations of low grade inflammation and endothelial dysfunction with depression–The Maastricht Study. *Brain, behavior, and immunity*. 2016;56:390-396.

© 2021 van der Heide FCT et al. *JAMA Network Open.*

Supplemental Figures

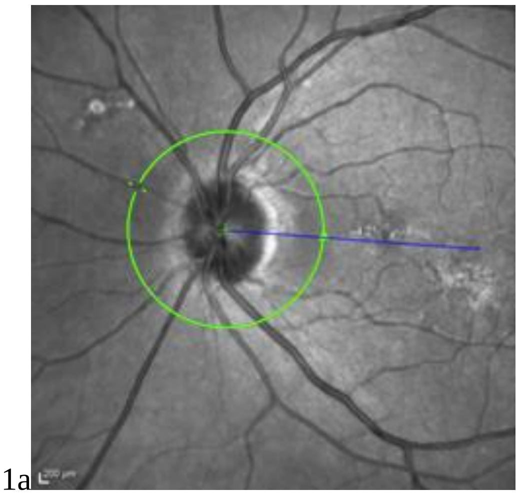

1a

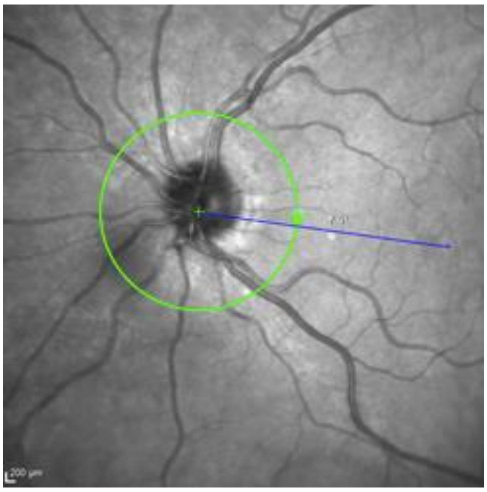

1b

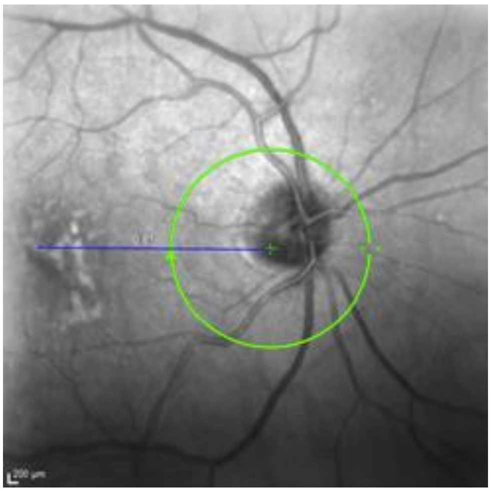

1c

eFigure 1. Examples of Quality of Centering of Circular Scans on the Optic Nerve Head

eFigure 1 shows examples of quality of centering of the circular scan on the optic nerve head: 1a shows good quality, 1b shows poor quality, and 1c shows very poor quality.

© 2021 van der Heide FCT et al. *JAMA Network Open.*

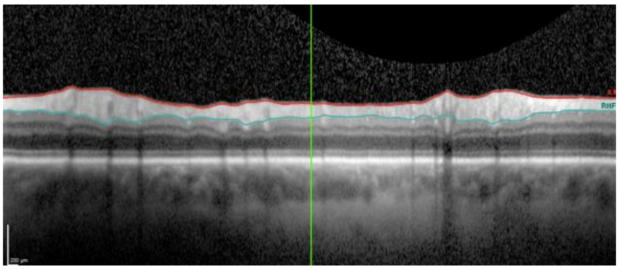

2a

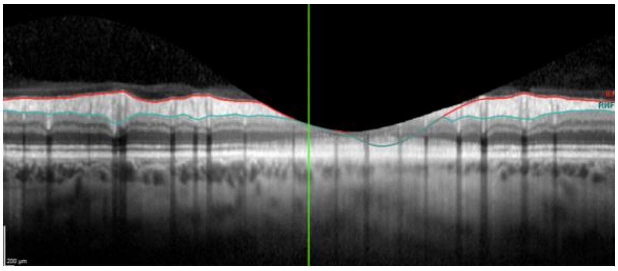

2b

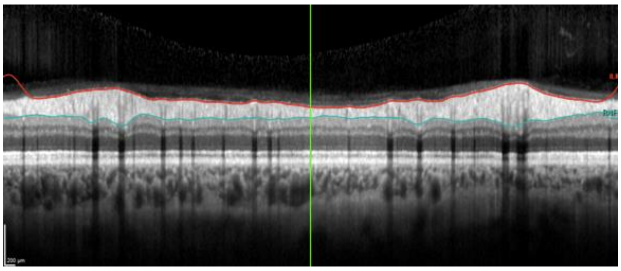

2c

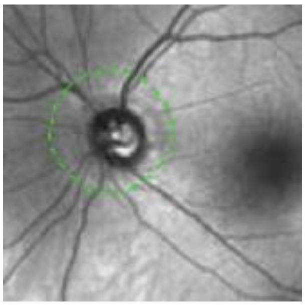

2d

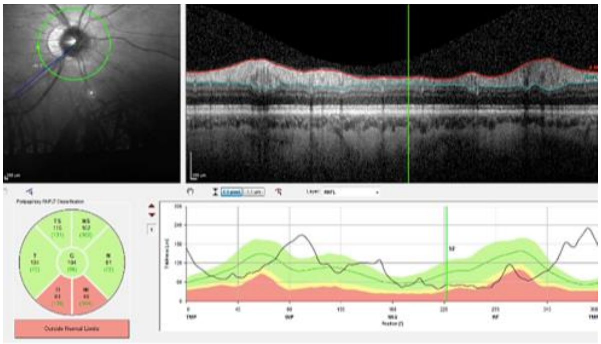

2e

© 2021 van der Heide FCT et al. *JAMA Network Open.*

2f 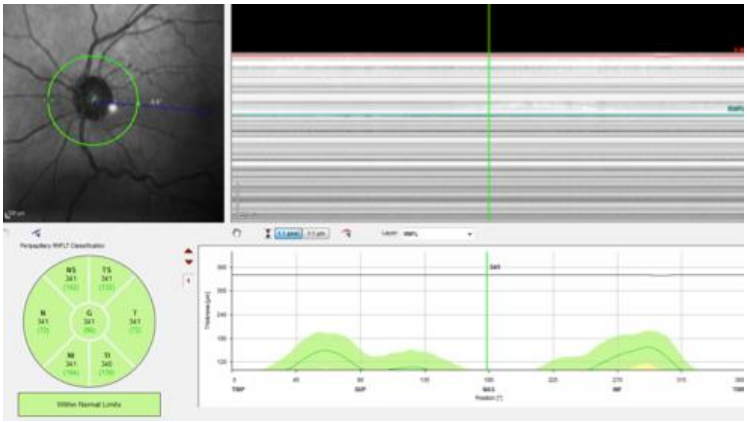

2g 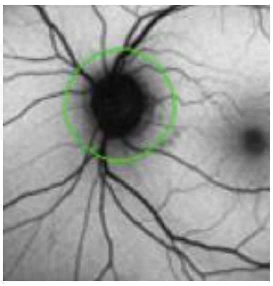

eFigure 2. Examples of Poor Imaging Quality and Scan Errors

2a: Example of poor imaging quality (Signal-to-noise ratio<15 dB); 2b: OCT device too close to the eye; 2c: RNFL layer incorrectly defined; 2d: incorrect circle position (dashed line); 2e: participant does not look in the correct direction; 2f: technical problem with OCT device; 2g: autofluorescence on.

Abbreviations: OCT, optical coherence tomography; RNFL, retinal nerve fiber layer thickness.

© 2021 van der Heide FCT et al. *JAMA Network Open.*

eTable 1. Additional General Characteristics of the Prospective Study Population With Complete Data on the Incidence of Clinically Relevant Depressive Symptoms, According to Tertiles of RNFL Thickness

Characteristic	Total study group (N = 4247)	Retinal nerve fiber layer thickness		
		Tertile 1 (low) (n = 1415)	Tertile 2 (middle) (n =1416)	Tertile 3 (high) (n = 1416)
Fasting plasma glucose (mmol/l) [a]	5.4 [5.0; 6.1]	5.4 [5.0; 6.3]	5.3 [4.9; 6.0]	5.3 [5.0; 6.0]
2-hour post load glucose (mmol/l) [a]	6.1 [4.9; 8.4]	6.3 [5.0; 9.0]	6.0 [4.9; 8.1]	6.0 [4.9; 8.0]
HbA1c (mmol/mol) [a]	37.0 [34.0; 41.0]	37.0 [34.0; 42.0]	37.0 [34.0; 40.5]	37.0 [34.0; 41.0]
Income level (euro) [a]	2078 ± 826	2092 ± 843	2089 ± 833	2045 ± 800
Occupational status [a]				
Low	381 (26.9)	129 (24.8)	120 (25.8)	132 (30.5)
Intermediate	542 (38.2)	185 (35.6)	188 (40.4)	169 (39.0)
High	495 (34.9)	206 (39.6)	157 (33.8)	132 (30.5)
Body mass index (kg/m2)	26.0 [23.7; 28.8]	26.2 [23.9; 28.9]	26.0 [23.5; 28.7]	26.0 [23.6; 28.8]
Systolic ambulatory blood pressure (mmHg) [a]	119.3 ± 11.4	120.2 ± 11.2	119.2 ± 11.4	118.5 ± 11.5
Diastolic ambulatory blood pressure (mmHg) [a]	73.8 ± 7.1	74.3 ± 6.9	73.8 ± 7.2	73.2 ± 7.1
Physical activity (hours/day) [a]	2.0 ± 0.7	2.00 ± 0.7	2.1 ± 0.7	2.0 ± 0.7
Dutch healthy diet score (points)*	84.5 ± 14.9	83.3 ± 14.7	84.9 ± 15.1	85.3 ± 14.7
Spherical equivalent (diopter) [a]	0.1 [-1.4; 1.1]	-0.6 [-3.0; 0.5]	0.1 [-1.2; 1.1]	0.5 [-0.4; 1.5]
Kidney variables [a]				
eGFR (ml/min)	82.0± 13.5	81.4 ± 13.7	81.7± 13.4	82.8 ± 13.4
Albuminuria				
Normoalbuminuria	3932 (92.8)	1298 (92.0)	1316 (93.3)	1318 (93.2)
Microalbuminuria	282 (6.7)	103 (7.3)	86 (6.1)	92 (6.5)
Macroalbuminuria	22 (0.5)	10 (0.7)	8 (0.6)	4 (0.3)
Prior cardiovascular disease [a]	662 (15.6)	240 (17.0)	213 (15.1)	209 (14.8)
Retinopathy*	55 (1.3)	19 (1.4)	13 (0.9)	23 (1.7)
Glaucoma	149 (4.5)	93 (7.0)	36 (2.7)	50 (3.7)
Intraocular pressure (mmHg) [a]	14.0 ± 3.0	14.4 ± 3.1	13.9 ± 2.9	13.8 ± 3.0
Plasma biomarkers of low-grade inflammation*				
C-reactive protein (µg/ml)	1.1 [0.6; 2.5]	1.2 [0.6; 2.7]	1.1 [0.6; 2.5]	1.1 [0.5; 2.4]
Serum amyloid A (µg/ml)	3.2 [2.0; 5.3]	3.3 [2.2; 5.6]	3.1 [2.0; 4.8]	3.2 [1.9; 5.4]
Interleukin-6 (pg/ml)	0.6 [0.4; 0.8]	0.6 [0.4; 1.0]	0.5 [0.4; 0.8]	0.5 [0.4; 0.8]
Interleukin-8 (pg/ml)	4.0 [3.2; 5.1]	4.2 [3.2; 5.3]	3.9 [3.2; 5.1]	3.9 [3.1; 4.8]
Tumor necrosis factor alpha (pg/ml)	2.2 [1.9; 2.5]	2.2 [1.9; 2.6]	2.1 [1.9; 2.5]	2.1 [1.8; 2.5]

Data are presented as n (%), mean ± standard deviation or median [interquartile range]

© 2021 van der Heide FCT et al. *JAMA Network Open.*

[a] Number of participants with data available for fasting plasma glucose, n= 4245; 2-hour post load glucose, n= 4068; HbA1c, n= 4243; income level, n= 3420; occupational status, n= 1418; office diastolic blood pressure, n= 4246; systolic ambulatory blood pressure, n= 3798; diastolic ambulatory blood pressure, n= 3798; physical activity, n= 3660; diet score, n= 4016; spherical equivalent, n= 4033; eGFR, n= 4244; albuminuria, n= 4236; prior cardiovascular disease, n= 4233; retinopathy, n= 4139; glaucoma, n= 4001; intraocular pressure, n= 4001; plasma biomarkers of low-grade inflammation, n= 1840.

Abbreviations: HbA1c, hemoglobin A1c; HDL, high-density lipoprotein; eGFR, estimated glomerular filtration rate; RNFL, retinal nerve fiber layer thickness.

© 2021 van der Heide FCT et al. *JAMA Network Open.*

eTable 2. General Characteristics of the Participants With Complete Data and Missing Data in the Prospective Study Population With Complete Data on the Incidence of Clinically Relevant Depressive Symptoms

**Characteristic, mean (SD)**	Participants with complete data (n=4432)	N missing data in participants with complete/ missing data	Participants with missing data (n=3573)
Age (years)	59.6 ± 8.4	0/0	60.1 ± 9.0
Women	2269 (51.2)	0/0	1715 (48.0)
Glucose metabolism status			
Type 2 diabetes	925 (20.9)	0/0	995 (27.8)
Other type of diabetes	20 (0.5)		30 (0.8)
Prediabetes	676 (15.3)		514 (14.4)
Normal glucose metabolism	2811 (63.4)		2034 (56.9)
Fasting plasma glucose (mmol/l)	5.4 [5.0; 6.1]	2/2	5.5 [5.0; 6.5]
2-hour post load glucose (mmol/l)	6.1 [4.9; 8.4]	197/312	6.2 [5.0; 9.1]
HbA1c (mmol/mol)	37.0 [34.0; 41.0]	4/10	38.0 [35.0; 44.0]
Educational status		0/116	
Low education	1457 (32.9)		1278 (37.0)
Medium education	1247 (28.1)		919 (26.6)
High education	1728 (39.0)		1260 (36.4)
Income level	2054 ± 827	873/1122	1942 ± 844
Occupational status			
Low	409 (27.9)	2964/2217	484 (35.7)
Intermediate	558 (38.0)		437 (32.2)
High	501 (34.1)		435 (32.1)
Waist circumference (cm)	94.1 ± 13.1	0/5	96.7 ± 14.2
Body mass index (kg/m2)	26.7 [24.2; 30.0]	0/3	26.7 [24.2; 30.0]
Total-to-HDL cholesterol ratio	3.3 [2.8; 4.1]	0/4	3.5 [2.8; 4.4]
Use of lipid-modifying medication	1314 (29.6)	0/6	1237 (34.7)
Office systolic blood pressure (mmHg)	133.1 ± 17.7	0/3	134.3 ± 18.2
Office diastolic blood pressure (mmHg)	75.5 ± 9.8	1/3	75.5 ± 9.8
Systolic ambulatory blood pressure (mmHg)	119.3 ± 7.1	476/609	119.9 ± 11.9
Diastolic ambulatory blood pressure (mmHg)	73.8 ± 7.1	476/609	74.0 ± 7.4
Use of antihypertensive medication	1566 (35.3)	0/6	1456 (40.8)
Smoking status		0/65	
Non-smoker	1714 (38.7)		1245 (35.5)

© 2021 van der Heide FCT et al. *JAMA Network Open.*

Former smoker	2211 (49.9)		1708 (48.7)
Current smoker	507 (11.4)		555 (15.8)
Alcohol consumption		0/66	
None	742 (16.7)		731 (20.8)
Low	2599 (58.6)		2029 (57.9)
High	1091 (24.6)		747 (21.3)
Partner status (with partner)	3635 (82.0)	0/58	2686 (76.4)
Physical activity (hours/day)	2.0 ± 0.7	617/657	1.9 ± 0.7
Dutch healthy diet score (points)	84.4 ± 14.9	253/431	83.0 ± 15.4
Spherical equivalent (diopter)	0.1 [-1.4; 1.1]	218/416	0.1 [-1.6; 1.1]
Use of antidepressants	290 (6.5)	0/6	287 (8.0)
Kidney variables			
eGFR (ml/min)	82.0 ± 13.6	3/5	82.5 ± 15.0
Albuminuria		13/74	
Normoalbuminuria	4097 (92.7)		3157 (90.2)
Microalbuminuria	299 (6.8)		309 (8.8)
Macroalbuminuria	23 (0.5)		33 (0.9)
Prior cardiovascular disease	699 (15.8)	15/86	638 (18.3)
Retinopathy	57 (1.3)	111/420	68 (2.2)
Glaucoma	187 (4.5)	251/444	148 (4.7)
Intraocular pressure (mmHg)	14.03 ± 3.04	251/444	13.9 ± 3.1
Plasma biomarkers of low-grade inflammation			
C-reactive protein (µg/ml)	1.1 [0.6; 2.6]	2521/1718	1.1 [0.7; 3.0]
Serum amyloid A (µg/ml)	3.2 [2.0; 5.3]	2521/1718	3.4 [2.1; 5.5]
Interleukin-6 (pg/ml)	0.6 [0.4; 0.9]	2521/1718	0.6 [0.4; 1.0]
Interleukin-8 (pg/ml)	4.0 [3.2; 5.2]	2521/1719	4.3 [3.4; 5.6]
Tumor necrosis factor alpha (pg/ml)	2.2 [1.9; 2.5]	2521/1718	2.2 [1.9; 2.6]
Peripapillary retinal nerve fiber layer thickness (µm)	94.8 ± 10.9 [a]	0/2454	94.7 ± 10.8 [a]
Depressive symptoms 1 year after baseline	2 [0; 4]	132/1920	2 [0; 4]
Depressive symptoms 2 years after baseline	2 [0; 4]	550/2195	2 [0; 4]
Depressive symptoms 3 years after baseline	2 [0; 4]	883/2317	2 [0; 4]
Depressive symptoms 4 years after baseline	2 [0; 4]	1253/2514	2 [0; 4]

© 2021 van der Heide FCT et al. *JAMA Network Open.*

Depressive symptoms 5 years after baseline	2 [0; 4]	1869/2607	2 [0; 4]
Depressive symptoms 6 years after baseline	2 [0; 4]	2950/3048	2 [0; 4]
Depressive symptoms 7 years after baseline	2 [0; 4]	3822/3320	2 [0; 4]
Incident clinically relevant depressive symptoms [b]	567 (12.8)	0†/1716	250 (13.5)
Median follow-up time (years)	5.0 [3.0-6.0]	0/2306	4.9 [2.1-6.0]

Data are presented as n (%), mean ± standard deviation or median [interquartile range]. Bold indicates P<0.05. Please note that the study population with complete data available (N=4432) differs in size from the prospective study population (N=4247) because for the main analyses participants with clinically relevant depressive symptoms (N=185) were excluded in the main analyses

[a] OCT scans of insufficient quality were not included in the calculation of the mean retinal nerve fiber layer thickness.

[b] n=3910 participants had complete data on incidence of clinically relevant depressive symptoms at all annual assessments and respectively n=444, n=66, and n=12 participants had missing data for one, two, or three assessments of clinically relevant depressive symptoms over time.

Abbreviations: HbA1c, hemoglobin A1c; HDL, high-density lipoprotein; eGFR, estimated glomerular filtration rate; OCT, optical coherence tomography.

© 2021 van der Heide FCT et al. *JAMA Network Open.*

eTable 3. *P* values of Interaction Terms of RNFL Thickness With Sex, Glucose Metabolism Status, or Time

		**Sex**	**Prediabetes**	**Type 2 diabetes**	**Time**
**Prospective**					
Incident clinically relevant depressive symptoms		0.60	0.48	0.94	0.06
Depressive symptoms		0.25	0.96	0.29	0.47

P-values for the interaction terms of sex, glucose metabolism status (i.e. prediabetes versus normal glucose metabolism status or type 2 diabetes versus normal glucose metabolism status), and time with RNFL thickness (e.g. sex*RNFL thickness). Variables in the model in addition to RNFL thickness and interaction term(s) with sex, glucose metabolism status and time are: age, sex, glucose metabolism status, educational level, waist circumference, total cholesterol/HDL ratio, use of lipid-modifying medication, office systolic blood pressure, use of antihypertensive medication, smoking status, alcohol consumption and partner status.

Bold indicates P<0.05.
Abbreviations: NA, not applicable; RNFL, retinal nerve fiber layer.

© 2021 van der Heide FCT et al. *JAMA Network Open.*

eTable 4. Cross-sectional Associations of Lower RNFL Thickness (per SD) With (Clinically Relevant) Depressive Symptoms or the Presence of a Major Depressive Disorder

		Crude	Model 1	Model 2
**Cross-sectional**		**RR (95% CI)**	**RR (95% CI)**	**RR (95% CI)**
**Depressive symptoms**		1.01 (0.98-1.05)	**1.04 (1.01-1.08)**	**1.04 (1.00-1.07)**
		**OR (95% CI)**	**OR (95% CI)**	**OR (95% CI)**
**Clinically relevant depressive symptoms**		0.94 (0.82-1.06)	0.96 (0.84-1.10)	0.96 (0.84-1.10)
**Major depressive disorder**		1.06 (0.90-1.24)	1.07 (0.91-1.26)	1.08 (0.92-1.27)

Regression coefficients represent the rate ratio or odds ratio (where applicable) for the presence of (clinically relevant) depressive symptoms or the presence of a major depressive disorder (cross-sectional analyses), per SD lower RNFL thickness, where a higher odds ratio or rate ratio indicates higher odds of the presence of clinically relevant depressive symptoms or the presence of a major depressive disorder, or higher rate ratio for more depressive symptoms. One SD of RNFL thickness corresponds with 10.86 micrometres in the cross-sectional (clinically relevant) depressive symptoms study population and 10.81 micrometres in the major depressive disorder study population.

Model 1: age, sex, glucose metabolism status, and educational status; Model 2: model 1 + waist circumference, total cholesterol / HDL cholesterol ratio, use of lipid-modifying medication, office systolic blood pressure, use of antihypertensive medication, smoking, alcohol consumption, and partner status.

Bold indicates P<0.05.

Abbreviations: CI, confidence interval; OR, odds ratio; RR, rate ratio; RNFL, retinal nerve fiber layer.

© 2021 van der Heide FCT et al. *JAMA Network Open.*

eTable 5. Associations of Lower RNFL Thickness (per SD) With the Incidence of Clinically Relevant Depressive Symptoms and Depressive Symptoms Over Time, Additional Analyses

	Crude	Model 1	Model 2
	**HR/RR (95% CI)**	**HR/RR (95% CI)**	**HR/RR (95% CI)**
**After exclusion of participants with use of anti-depressive medication at baseline**			
Incidence of clinically relevant depressive symptoms, HR (95%CI), n=4012	1.07 (0.97-1.18)	1.09 (0.98-1.21)	1.10 (0.99-1.22)
Depressive symptoms, RR (95%CI), n=4463	1.02 (0.99-1.04)	1.03 (1.01-1.06)	1.03 (1.01-1.06)
**After exclusion of participants with clinically relevant depressive symptoms and/or a major depressive disorder at baseline**			
Incidence of clinically relevant depressive symptoms, HR (95%CI), n=3913	**1.12 (1.01-1.23)**	**1.15 (1.04-1.27)**	**1.15 (1.04-1.27)**
Depressive symptoms, RR (95%CI), n=4526	1.02 (1.00-1.05)	**1.04 (1.02-1.07)**	**1.04 (1.02-1.07)**
**Without exclusion of participants with clinically relevant depressive symptoms at baseline**			
Incidence of clinically relevant depressive symptoms, HR (95%CI), n=4432	1.05 (0.96-1.14)	1.08 (0.99-1.17)	1.08 (1.00-1.18)
Depressive symptoms, RR (95%CI), n=5170	1.01 (0.99-1.04)	**1.03 (1.01-1.06)**	**1.03 (1.01-1.06)**
**After exclusion of participants without incident clinically relevant depressive symptoms when they had at least 2 missing assessments of PHQ-9 data over 7 follow-up measurements**			
Incidence of clinically relevant depressive symptoms, HR (95%CI), n=4231	1.08 (0.99-1.19)	**1.11 (1.01-1.22)**	**1.12 (1.01-1.23)**
Depressive symptoms, RR (95%CI), n=4160	1.00 (1.00-1.05)	1.02 (0.99-1.04)	1.02 (0.99-1.04)
**After exclusion of participants with an age of major depressive disorder onset of ≤ 40 years**			
Incidence of clinically relevant depressive symptoms, HR (95%CI), n=3755	1.08 (0.97-1.20)	1.09 (0.98-1.22)	1.10 (0.99-1.23)
Depressive symptoms, RR (95%CI), n=4350	1.02 (0.99-1.18)	**1.04 (1.01-1.07)**	**1.04 (1.01-1.07)**

© 2021 van der Heide FCT et al. *JAMA Network Open.*

Regression coefficients represent the hazard ratio for the incidence of clinically relevant depressive symptoms per SD lower RNFL thickness, where a higher hazard ratio or rate ratio indicates higher incidence of clinically relevant depressive symptoms or more depressive symptoms over time. In all models 1 SD corresponds with 10.9µm.

Model 1: age, sex, glucose metabolism status, and educational status; Model 2: model 1 + waist circumference, total cholesterol / HDL cholesterol ratio, use of lipid-modifying medication, office systolic blood pressure, use of antihypertensive medication, smoking, alcohol consumption, and partner status.

Bold indicates P<0.05.
Abbreviations: RR, rate ratio; HR, hazard ratio; CI, confidence interval; RNFL, retinal nerve fiber layer; PHQ-9, patient health questionnaire-9; NA, not applicable.

© 2021 van der Heide FCT et al. *JAMA Network Open.*

eTable 6. Associations of Lower RNFL Thickness (per SD) With the Incidence of Clinically Relevant Depressive Symptoms and Depressive Symptoms Over Time, Additionally Adjusted for Physical Activity (Model 3), Diet Score (Model 4), Spherical Equivalent (Model 5), Use of Antidepressive Medication (Model 6), Kidney Variables (Model 7), Prior Cardiovascular Disease (Model 8), Retinopathy (Model 9), Glaucoma (Model 10), and Plasma Biomarkers of Low-grade Inflammation (Model 11), or After Exclusion of Individuals With Retinopathy (Model 12), or Glaucoma (Model 13)

	**Incidence of clinically relevant depressive symptoms, HR (95%CI)**	**Depressive symptoms, RR (95%CI)**
**Model 3**	**1.12 (1.01-1.24)**	**1.04 (1.01-1.07)**
**Model 4** [a]	1.10 (1.00-1.22)	**1.04 (1.01-1.06)**
**Model 5**	**1.15 (1.03-1.27)**	**1.04 (1.02-1.07)**
**Model 6**	1.10 (1.00-1.21)	**1.03 (1.01-1.06)**
**Model 7**	**1.11 (1.01-1.22)**	**1.04 (1.01-1.06)**
**Model 8**	**1.12 (1.01-1.23)**	**1.04 (1.01-1.06)**
**Model 9**	**1.12 (1.01-1.23)**	**1.04 (1.01-1.06)**
**Model 10**	**1.15 (1.04-1.28)**	**1.05 (1.02-1.07)**
**Model 11**	1.13 (0.97-1.30)	**1.04 (1.01-1.08)**
**Model 12**	**1.13 (1.02-1.24)**	**1.04 (1.01-1.07)**
**Model 13**	**1.16 (1.05-1.28)**	**1.05 (1.02-1.08)**

Regression coefficients represent hazard ratio for incidence of depressive symptoms or rate ratio for depressive symptoms over time per SD lower RNFL thickness, where a higher hazard ratio or rate ratio indicates higher incidence of clinically relevant depressive symptoms or more depressive symptoms over time. One SD of RNFL thickness corresponds with 10.9 micrometres in the incidence of clinically relevant depressive symptoms study population in model 3 and 11.0 micrometres in the depressive symptoms over time study population. In other models the value per SD was numerically comparable.

Variables entered in all models : age, sex, and glucose metabolism status, educational status, waist circumference, total cholesterol / HDL cholesterol ratio, use of lipid-modifying medication, office systolic blood pressure, use of antihypertensive medication, smoking, alcohol consumption, and partner status.

[a] Model 4 was adjusted for the 'adjusted dietary intake score'(i.e. dietary intake score without alcohol consumption as part of the dietary intake score).

Number of participants with respectively complete data for for incidence of depressive symptoms or depressive symptoms over time : model 3, n=3658 and n=4242; model 4, n=4012 and n=4620; model 5, n=4029 and n=4683; model 6, n=4247 and n=4934; model 7, n=4229 and n=4913; model 8, n=4229 and n=4916; model 9, n=4136 and n=4809; model 10, n=3997 and n=4641; model 11, n=1836 and n=2151; model 12, n=4970 and n=4742; model l3, n=3822 and n=4429.

Bold indicates P<0.05.

Abbreviations: RR, rate ratio; HR, hazard ratio; CI, confidence interval; RNFL, retinal nerve fiber layer.

© 2021 van der Heide FCT et al. *JAMA Network Open.*

eTable 7. Associations of Lower RNFL Thickness (per SD) With the Incidence of Clinically Relevant Depressive Symptoms and Depressive Symptoms Over Time, Replacement of Glucose Metabolism Status With Fasting Plasma Glucose (Model 3A), 2-hour Post Load Glucose (Model 3B) or Hb$_{A1c}$ (Model 3C), Waist Circumference With BMI (Model 3D), Office Systolic Blood Pressure With Office Diastolic Blood Pressure (Model 3E), 24-hour Ambulatory Systolic Blood Pressure (Model 3F) and 24-hour Ambulatory Diastolic Blood Pressure (Model 3G), and Educational Status With Income Level (Model 3H) or Occupational Status (Model 3I)

	**Incident clinically relevant depressive symptoms, HR (95%CI)**	**Depressive symptoms RR (95%CI)**
**Model 3A**	**1.11 (1.01-1.23)**	**1.04 (1.01-1.06)**
**Model 3B**	1.10 (1.00-1.22)	**1.04 (1.01-1.06)**
**Model 3C**	**1.11 (1.01-1.23)**	**1.04 (1.01-1.06)**
**Model 3D**	**1.11 (1.01-1.23)**	**1.04 (1.01-1.06)**
**Model 3E**	**1.11 (1.01-1.22)**	**1.04 (1.01-1.06)**
**Model 3F**	**1.13 (1.02-1.25)**	**1.03 (1.01-1.06)**
**Model 3G**	**1.13 (1.02-1.25)**	**1.03 (1.01-1.06)**
**Model 3H**	1.04 (0.93-1.16)	1.04 (1.00-1.08)
**Model 3I**	1.05 (0.88-1.26)	1.01 (0.97-1.06)

Regression coefficients represent hazard ratio for incidence of depressive symptoms or rate ratio for depressive symptoms over time per SD lower RNFL thickness, where a higher hazard ratio or rate ratio indicates higher incidence of clinically relevant depressive symptoms or more depressive symptoms over time. One SD of RNFL thickness corresponds with 10.9 micrometres in the incidence of clinically relevant depressive symptoms study population in model 3A and 11.0 micrometres in the depressive symptoms over time study population, and. In other models the value per SD was numerically comparable.

Variables in models 3A-4I: age, sex, and glucose metabolism status (where applicable), educational status (where applicable), waist circumference, total cholesterol / HDL cholesterol ratio, use of lipid-modifying medication, office systolic blood pressure (where applicable), use of antihypertensive medication, smoking, alcohol consumption, and partner status.

Number of participants with respectively complete data for for incidence of depressive symptoms or depressive symptoms over time: model 3A, n=4241 and n=4932; model 3B, n=4065 and n=4711; model 3C, n=4239 and n=4929; model 3D, n=4243 and n=4934; model 3E, n=4242 and n=4933; model 3F, n=3795 and n=4380; model 3G, n=3795 and n=4380; model 3H, n=3420 and n=1654,; model 3I, n=1177 and n=1362.

Bold indicates P<0.05.
Abbreviations: RR, rate ratio; HR, hazard ratio; CI, confidence interval; RNFL, retinal nerve fiber layer.

© 2021 van der Heide FCT et al. *JAMA Network Open.*
